# Self-perception of occupational stigma among nurses – experience from Chinese nurses: a descriptive phenomenological research study

**DOI:** 10.1186/s12912-026-05023-x

**Published:** 2026-07-11

**Authors:** Dexiu Yan, Yuzhen Zhou, Jian Chang, Yuanyuan Zhang, Lan Chen

**Affiliations:** 1 Department of Nursing, Shanghai General Hospital, Shanghai, China; 2 Shanghai Jiao Tong University School of Nursing, Shanghai, China

**Keywords:** Occupational stigma, Nurse, Qualitative study

## Abstract

**Objective:**

This research aims to understanding of nurses’ self-perceptions of professionalism and to explore their professional experiences in the workplace. It particularly focuses on the impact of occupational stigma—a key issue in nursing and mental health.

**Background:**

Occupational perceptions significantly influence nurses’ attitudes toward work. Understanding their professional experiences and social interactions is essential. Examining the impact of occupational stigma also promotes their occupational mental health.

**Methods:**

We employed a phenomenological research methodology. We used a semi-structured interview question framework to gain a deep understanding of nurses’ experiences at work, particularly their self-perceptions of their profession. From August 15, 2023, to October 20, 2023, we recruited 14 study participants from various departments in Shanghai, China. We conducted the interviews in their native language. The content of the interviews was analysed using Colaizzi’s phenomenological approach.

**Results:**

The study identified three main themes: physical stigma (three sub-themes), social stigma (three sub-themes), and ambivalent self-identity (three sub-themes).

**Conclusion:**

Nursing work, due to its nature and hazards, can evoke a sense of physical ‘stigma’ among practitioners. This highlights the need for nurses to prioritise occupational protection. It also calls for managers and health authorities to raise awareness. However, public bias and misconceptions about nursing show the need for positive media and government advocacy. Interestingly, nurses’ perceptions of their profession are often contradictory. They experience both the dangers and advantages associated with the role. This creates tension between their recognition of the profession’s importance and possible denial of it due to pressure and risk. By improving occupational protection and providing positive guidance, managers can help nurses strengthen their professional identity, reduce occupational denial, and ensure the nursing profession develops stably.

## Background

Globally, nurses play a central role in healthcare systems, providing direct nursing care, health education, and support to patients and their families [1]. According to the World Health Organisation (WHO), nurses comprise more than 50 per cent of the global health workforce [2]. The nursing profession is often perceived as a female-dominated field [3] and as a “woman’s vocation”, which can lead to its undervaluation [4]. In particular, male nurses tend to suffer more discrimination and prejudice, with their motivation and ability to make career choices frequently questioned [5]. In addition, nurses are often portrayed in films, television dramas, and news reports as doctors’ assistants, needing more ability to make independent decisions. These stereotypes undermine public recognition of nurses’ professional competence, leading to the underestimation of their roles and contributions, which are understood and more highly valued, despite their enormous contributions to public health.

Furthermore, these biases often shape nurses’ professional experiences in the workplace, influencing their sense of self-worth and their interactions with patients. To ensure that nurses can fulfil their roles in a fair and respectful environment and contribute more effectively to healthcare development, addressing the occupational stigma they face is urgently needed [6]. Hughes [7] (1951) first introduced the term “non-decent work” (also known as “dirty” work) to describe occupations considered to be of low social status and often involving heavy, messy, or hard work, such as cleaners. This term highlights that certain jobs may be evaluated negatively at both the social and cultural levels [8]. Organisational behaviour scholars define occupational stigma as the extent to which a profession is looked down upon and excluded by the public because of certain “stigmatising” characteristics [9]. For practitioners, the sense of occupational stigma can be viewed as one of the sources of pressure they face both inside and outside the workplace [10]. Nurses’ occupational stigma is a significant issue, including the negative labels and evaluations that the public assigns to nurses’ behaviours, characteristics, social status, and the nurse-patient relationship [11]. It’s crucial to recognise that the public, including us as healthcare professionals, plays a major role in perpetuating this stigma. Understanding this role is key to addressing and overcoming it.

For individuals, stigma is a complex interplay of cognition, emotion, and behaviour. Cognitions reflect nurses’ negative public perceptions and evaluations [11], while emotional and behavioural responses can reinforce negative beliefs about them. For instance, if a nurse’s attitude toward a patient is perceived as indifferent, the patient may develop resentment, leading to verbal abuse or even irrational violence. These public perceptions and reactions significantly shape nurses’ professional experiences in the workplace. Stigma affects their interactions with patients, colleagues, and the broader healthcare environment, diminishing their job satisfaction and well-being. The impact of this stigma on nurses’ well-being is a cause for concern and underscores the need for its immediate address.

Although public awareness of nurses’ professionalism and importance has increased with the growing demand for healthcare, occupational stigma persists [12]. During the Reformation, nursing was carried out by untrained and inexperienced individuals [13], and nurses were often associated with negative labels such as ‘lowly servant’ and ‘vulgar’ [14]. Nursing was also considered a humble profession. It was not until the Crimean War, when Florence Nightingale’s professionalism and dedication to improving healthcare in military hospitals were recognised, that the status of nurses was elevated and they came to be viewed as ‘angels in white’ [15]. However, currently, this historical legacy has been undermined, as nurses—particularly those working night shifts, interacting with strangers, or performing ‘dirty’ tasks—are often stigmatised [16]. For example, nurses in emergency departments frequently deal with death, dress wounds, and handle vomit, which may result in them being shunned [17].

The pervasive occupational stigma against nurses has far-reaching implications for healthcare. It not only undermines the professional standing of nurses but also deters top talent and affects the quality of healthcare delivery [18]. While existing research has examined the occupational stigma experienced by specific groups of nurses, such as male nurses [5], mental illness nurses [19], and emergency department nurses [20], there is a pressing need for a more comprehensive understanding of nurses’ professional experiences in general hospitals. This study aims to examine nurses’ self-perceptions of occupational stigma in general hospitals. To achieve this, a qualitative research methodology will be employed. This approach will allow us to explore nurses’ professional experiences and how stigma influences their work environments and interactions.

## Methodology

### Design and procedure

This study adopted a hermeneutic phenomenology (Heidegger) research methodology rooted in individual experience [21] to investigate nurses’ subjective understanding of their nursing care. The phenomenological description method allows for a deep exploration of participants’ experiences and perspectives, making it particularly suited to areas with limited prior research. The researcher received meticulous training in qualitative research methods, holds a PhD in nursing, and is research-qualified to conduct interviews. Also, the researcher has conducted phenomenological research and has the appropriate research skills.

### Sample recruitment

Participants were recruited using purposive sampling from a comprehensive grade A hospital in Shanghai, which provides care for patients with various diseases. In China, nurses in tertiary hospitals are primarily responsible for a range of tasks, including taking over and handing over shifts, processing medical prescriptions, administering treatments, providing quality nursing care, and managing medications. Nurses are paid differently depending on the department they work in, and night shift nurses are paid extra. However, due to China’s large population, the nurse-to-bed ratio is less than 1:0.8, resulting in a higher occupational burden for nurses.

To ensure maximum diversity in the sample, participants were selected from different medical departments, including Emergency, ICU, Pediatrics, Obstetrics and Gynaecology, Internal Medicine, Surgery, Hemodialysis Unit, and Outpatient Department.

Inclusion criteria were as follows: (a) Registered nurses with valid professional certification, (b) Engaged in clinical nursing for more than 1 year, and have the ability to generate clear career awareness. (c) Willingness to participate in the study and sign an informed consent form. Participant recruitment was conducted solely by one researcher.

### Ethics

This study received approval from the Ethics Committee of the XXX Hospital of Shanghai. The initial study participant was referred by a researcher’s friend (the researcher did not know the participant well), while subsequent participants were recruited via snowball sampling. Prior to the interviews, the researcher had secured the participants’ WeChat accounts (a free messaging and phone application widely used in China) through the referrer and contacted them to inform them of the purpose of the interview. During the process of engaging with the participants, the researcher confirmed the interview method, timing, and location, avoiding any discussion of the study’s specific details, and thus ensuring the participants’ voluntary participation.

With the participants’ consent, the researchers recorded the interview time and audio to collect data. Transcription of the recordings was completed within 48 h after the interview. The researchers provided the written material to each participant. If participants had questions or provided new information, they could contact the researchers at any time. The researchers would then engage in further discussions and clarifications until a consensus was reached. Participants had the right to stop participating in the study or withdraw from it at any time without obligation or consequence. Participants who chose not to participate faced no penalties or loss of benefits.

### Data collection procedures

From August 15, 2023, to October 20, 2023, we conducted in-depth interviews using a semi-structured interview guide (see Table 1). The outline for the semi-structured interviews was based on the findings from interviews with two pre-experimental study participants. Recognising that some participants needed to balance work and family responsibilities, the researchers offered participants the option to choose between the two interview formats. On average, the interviews lasted for 35 min. Following the interviews, each participant’s conversation was transcribed verbatim. The entire process was conducted in the participants’ and interviewers’ native language, which is Chinese.

**Table 1:** Semi-structured question

Semi-structured question
1. How do you perceive the social status of nurses?
2. What relevant experiences do you have in your work?
3. How have these experiences affected you?
4. How do the patients or doctors you interact with evaluate nurses?
5. How have these evaluations affected you?
6. How do your family members evaluate your work?
7. How have these evaluations affected you?
8. What do you think is the value of nursing work?

### Data analysis

The data analysis was performed utilising NVivo 12 software, which employs Colaizzi’s phenomenological analysis technique [22] (Colaizzi, 1978). Throughout the analytical process, NVivo 12 facilitated the creation of a markup index, allowing the researcher to record thoughts directly within each code detail page. Additionally, the researcher shared codes and their descriptions with research team members for informal peer debriefing, thereby ensuring the effective execution of Bracketing. Colaizzi’s seven-step phenomenological analysis method offered a structured framework for an exhaustive examination of the data, encompassing: (a) A meticulous reading of all data. (b) The identification of significant statements. (c) The coding of recurring themes. (d) The aggregation of coded themes. (e) The composition of a detailed description. (f) The differentiation of similar themes. (g) The process of returning to participants for validation.

Lincoln and Guba’s criteria [23] (Guba, 1985) were employed to assess the qualitative research and ensure trustworthiness. The researcher’s scientific experience and two-person analysis ensured the study’s intrinsic validity. Finally, member checking was conducted to clarify information mentioned in the translation of the data, including the translation and back-translation. The translation of the data was started by two studies separately, collated and integrated by another study member, and, after a meticulous discussion, resulted in a translated manuscript, which in turn was invited to be back-translated by someone with study-abroad experience in the United States. After comparing the back-translated version with the original, the differences between the translated and original versions were small, and the translated version of this study was able to formally present the representations of the research participants.

## Results

### Characteristics of subjects

This study recruited 14 participants, with interview durations ranging from 31 to 65 min. The participants consisted of 13 females and 1 male, aged 23 to 44. General information about the participants can be found in Table 2. According to the Colaizzi phenomenological analysis method, this study identified three main themes: “Physical stigma,” “Social stigma,” and “Ambivalent self-identity,” along with 9 sub-themes (See Table 3).

**Table 2:** Social demographic of nurses

Serial number	Gender	Age	Experience years	Position	Title	Department	Education
N1	Female	30	9	Clinical Nurse	Nurse Practitioner	Hematology	Bachelor
N2	Male	30	2	Clinical Nurse	Nurse Practitioner	Hemodialysis Laboratory	Master
N3	Female	28	6	Clinical Nurse	Nurse Practitioner	Oral Surgery	Graduate Students
N4	Female	26	4	Clinical Nurse	Nurse Practitioner	ICU	Undergraduate
N5	Female	36	13	Nurse Manager	Charge Nurse	Pediatrics	Undergraduate
N6	Female	35	13	Clinical Teaching	Nurse Practitioner	Geriatrics	Undergraduate
N7	Female	36	10	Nurse Specialist	Charge Nurse	Assisted Reproduction	Undergraduate
N8	Female	23	3	Clinical Nurse	Nurse Practitioner	Operating Room	Specialized
N9	Female	25	3	Clinical Nurse	Nurse Practitioner	Emergency	Undergraduate
N10	Female	33	10	Clinical Teaching	Charge Nurse	Thoracic Surgery	Undergraduate
N11	Female	30	8	Clinical Nurse	Nurse Practitioner	Stomatology	Undergraduate
N12	Female	25	3	Clinical Nurse	Nurse Practitioner	Operating Room	Undergraduate
N13	Female	26	3	Clinical Nurse	Nurse Practitioner	Neurosurgery	Undergraduate
N14	Female	44	20	Director of Nursing	Associate Nurse	Nursing	PhD student

**Table 3:** The themes of this study

Themes	Sub-themes
Physical stigma	Excessive physical strain
	Occupational exposure risks
	Emotional exhaustion
Social stigma	Low social status
	Lack of recognition and respect
	Subject to public criticism and misunderstanding
Ambivalent self-identity	Positive Perceptions of the Profession
	Negative Experiences in the Profession
	Contributing to “Intent to Leave”

## Physical stigma

### Excessive physical strain

Participants indicated that their work is completed amid various sources of contamination and requires high levels of tension and pressure. Nursing as a profession is highly specialised and requires practitioners to possess solid medical knowledge and the ability to handle a range of emergencies. Nonetheless, nursing staff remain on the front line, driven by a love for their profession and a sense of responsibility to their patients, providing care and support.

N2: There is a Chinese saying that ‘A doctor says a word, and a nurse breaks her leg’. Compared with technical work, I think nurses are more inclined to be manual workers.

N4: There are always a lot of emergency devices constantly alarming at work, and you need to run before each device all the time, and occasionally there are sudden resuscitations, and you need to stay tight at work all the time.

### Occupational exposure risks

In hospital settings, healthcare workers encounter many patients and complex medical conditions every day and inevitably come into contact with bacteria, viruses, and other pathogens, which pose ongoing health risks.

N6: We had a colleague who had experienced a needlestick injury and was unfortunately infected, and for a long time, I was afraid that I would be threatened by this as well.

N14: Not long ago, my colleagues were beaten by patients. We didn’t do anything wrong, but we were attacked for no reason. I don’t think other jobs should face this danger.

### Emotional exhaustion

1. Passive labor.

As passive workers, nurses are often in a reactive work situation. Their duties require them to be on call at all times and to respond quickly to a wide range of patient needs, assisting with treatment and care as directed by doctors. The pace of work is usually dictated by changes in a patient’s condition, emergencies, and the doctor’s schedule, which means they can rarely organise their workload autonomously and often struggle to respond to emergencies and unpredictable workloads. On top of this, nurses must always be ready to provide timely help and care to patients, both during the day and at night. This high level of passivity and stress makes nursing a challenging profession.

N10: Our work is mainly carried out around the doctor’s advice. As for what drugs patients use and what examinations they need to do, it is decided by doctors. We have no autonomy in our work. Even if we sometimes find a mistake, we need to be corrected by the doctor before carrying it out.

N9: In our work, we are often seen as mere executors of orders—whether it’s preparing medications or assisting with procedures. Even when we notice something amiss, we are taught to wait for the doctor’s approval before taking action, leaving little room for our expertise to be fully acknowledged.

(2) Negative Energy.

The nursing work environment is often full of negative energy, primarily due to the constant exposure to patients’ pain, suffering, and anxiety. Nurses are regularly confronted with the emotional and physical distress of those they care for. The cumulative effect of these factors places nurses under immense psychological stress, as they must not only manage their own emotions but also provide comfort and support to those around them.

N3: Many of the patients we receive in our unit have gone through a very torturous experience of seeking medical treatment, which leads to a lot of negative emotions when they come to the hospital, and some of the illnesses are with them for a long time, which leads to a poor mood for patients and dependents.

N7: The patients who come here for treatment are accompanied by fertility problems, and their own psychology is very fragile, and they accumulate very many negative emotions when they fail to have children, and the whole treatment period is very depressing.

N1: The patients we care for often come with deep emotional burdens—be it from prolonged illnesses, fertility struggles, or the anxiety of post-surgery recovery. Their pain and frustration can be overwhelming, and when families share in that stress, it’s hard to maintain an atmosphere of calm and respect.

(3) A heavy emotional burden.

The nursing profession requires significant emotional resilience, as nurses frequently encounter negative emotions stemming from patients’ pain, families’ anxieties, and the high demands. Nurses must deal with these emotional situations daily, often comforting patients and families while managing their own emotional responses. In addition to providing compassionate care, nurses often shoulder responsibilities such as addressing administrative concerns and managing misunderstandings that can lead to frustration and complaints, further adding to their emotional burden. Despite these challenges, nurses remain committed to providing compassionate care, often prioritising the emotional well-being of others over their own.

N5: In addition to our own work, we also need to be responsible for a lot of work. For example, if a patient finds something wrong with the expense list, he will not go to the financial department to ask; he will be accountable to you first. When it comes to money, the patient is often very distressed.

N11: Every Monday is the peak period for patients to be discharged from the hospital, but there is no special personnel to assist patients to go through the corresponding formalities, which leads to all people coming to consult us. However, if some of them are not explained clearly, patients will feel that there is something wrong with your work and complain against us.

### Social stigma

#### Low social status

The study participants generally expressed concern about the low social status of nurses, viewing it as a significant issue requiring urgent attention and resolution. Despite their vital role in patient care and the healthcare system, nurses often find their contributions undervalued and their profession afforded less respect and prestige than other medical roles. This low social status can affect not only nurses’ morale but also the profession’s perception, potentially discouraging new talent from entering the field.

N7: When it comes to the social status of nurses, the profession of nurses is not popular with men in the marriage market. I think this is also an indication of the low social status of nurses.

N10: In contrast, patients are more willing to listen to the doctor’s opinion, even if what we say is the same. In many cases, I need to call the doctor to confirm what I said.

N3: When drawing blood from patients, they will question me about the use of the blood drawn, and even say that we want to steal it, but they don’t question anything when the doctor checks in.

### Lack of recognition and respect

Despite nurses’ critical role in the healthcare system, their professional status often lacks recognition and respect. Nurses are essential to patient care, providing medical support, emotional comfort, and advocacy for their patients. However, their contributions are sometimes undervalued, both within the healthcare hierarchy and by society at large.

N1: I can’t feel the respect of patients for us. This respect is not reverence for the profession, but rather the most common form of respect between people. Patients often yell at us, and their attitude towards us is not even as good as that of hotel waiters.

N6: There is an older veteran cadre who told me very clearly that nurses are not worthy of communicating with him, which is even insulting enough.

N4: ’Your work isn’t vital’, ‘Resuscitation isn’t your business, it’s the doctor’s’, even my family can’t understand the value of my work.

### Subject to public criticism and misunderstanding

1. Misconceptions About Professional Skills.

Nurses often have to accept negative evaluations, which can stem from misunderstandings about their job responsibilities and professional abilities. These negative evaluations are not limited to patients and their families; even some doctors may contribute to this dynamic. Misunderstandings about the scope of nursing practice, coupled with the high-pressure healthcare environment, can undervalue nurses’ expertise and contributions.

N12: When the patient has a disease change, the doctor will consider our responsibility first. For example, there is a change in the patient’s blood indicators, he will first consider whether the nurse made an error, not the patient’s own change.

N14: When talking about nurses, people mainly think of low education, poor attitude and strong substitutability. But the real situation is not like that. We have experienced a lot of training and learned a lot of professional knowledge. Many colleagues have graduated from famous schools, but patients still evaluate us with their inherent thinking.

(2) Facing public criticism.

Nurses are typically the members of the medical team who have the closest and most frequent contact with patients. This proximity makes them the most visible representatives of the healthcare system in the public’s eyes. As a result, any dissatisfaction, frustration, or misgivings that patients or their families may have about the healthcare system are often directed toward the nurses they interact with the most. Whether these issues stem from broader systemic problems, such as long wait times, resource shortages, or the outcomes of medical treatments, nurses can find themselves bearing the brunt of public dissatisfaction.

N3: In times of conflict between doctors and patients, nurses are often the ones who bear the brunt of the anger. When patients are unhappy with their treatment or the hospital services, they frequently direct their frustration at us, blaming us for everything from medical costs to perceived neglect. It’s as if we’re the easiest target, even though we’re not making the critical decisions.

N6: When patients are dissatisfied with the diagnosis and treatment plan or hospital services, they will vent their grievances on nurses, especially in the process of nursing operations. Patients often accuse nurses of only knowing that fees do not save people.

### Ambivalent self-identity

#### Positive perceptions of the profession

1. Technologically advanced.

Participants pointed out that nursing is a highly technical profession, far more complex than typical work in the general service industry. Nurses must possess a deep understanding of advanced medical equipment, from diagnostic machines to life-support systems, and be able to operate these tools precisely. In addition to their technical expertise, nurses must skillfully navigate various medical software platforms for patient records, medication management, and treatment planning.

N8: I think the professional value of nurses is not only reflected in the basic nursing content, especially the nurses in the operating room. We need to assist doctors to carry out operations, which requires very strong professionalism.

N13: Our hospital is also carrying out nursing clinics, such as wound clinics, peritoneal dialysis clinics, intravenous treatment clinics, especially PICC nursing clinics, and many patients come to see a doctor every day.

(2) Promoting social development.

Nursing work is crucial in promoting social development by serving as the first defence in protecting and enhancing public health. Nursing work effectively reduces the burden on hospitals and medical institutions by providing comprehensive care and support. This alleviation allows doctors to focus more on complex and critical medical problems, thereby improving the overall efficiency and effectiveness of the healthcare system. Additionally, by managing routine and preventive care, nurses contribute to better health outcomes and quality of life, fostering a healthier population and supporting broader social development goals. Their role enhances individual patient well-being and strengthens the healthcare infrastructure, benefiting society.

N13: Nurses did a lot of work during the epidemic. Basically, every nurse participated in the internal or external support of the hospital and played a very important role in maintaining the operation of medical institutions.

N9: In addition to the basic nursing services seen by patients, nurses also work in many positions. For example, our triage nurses in emergency department have played a very important role in handling batch trauma rescue and pretreatment of patients.

(3) Job Stability.

The nursing field offers relatively stable salaries and job security compared to many other industries. This stability is partly due to the increasing demand for healthcare services, particularly as the ageing population grows. The need for nursing care is projected to rise, ensuring a consistent demand for skilled professionals. Also, nursing is considered a resilient profession that remains in demand despite economic fluctuations. Whether during economic downturns or periods of growth, the essential nature of nursing care remains significant, contributing to the profession’s overall occupational stability. This stability provides financial security for nurses and offers a sense of job reliability and long-term career prospects in a profession dedicated to improving public health.

N1: In recent years, the world has entered a financial crisis, and employment forms are severe. Many large enterprises have begun laying off employees. During the epidemic, many people lost their economic resources. In contrast, the employment status and occupational stability of the nursing industry are very good.

N10: Although nursing work is hard, it is cost-effective. Hospitals are public institutions; nurses have a fixed establishment and personnel system, and their positions are relatively stable and not easily affected by dismissal or layoffs. In addition, our salary is usually relatively fair, and the salary level and promotion channel are relatively open and transparent.

#### Negative experiences in the profession

1. Lack of family support.

Family disapproval is a significant challenge faced by many nurses, particularly in cultures where traditional gender roles and expectations influence career choices. In professions like nursing, characterised by low social status, long working hours, shift systems, and high stress, the alignment with conventional views of women’s roles—such as “caring for each other and teaching children”—can be seen as lacking. This misalignment often leads to a lack of recognition and support from family members, who may not fully appreciate the demands and contributions of the nursing profession. In traditional contexts, where familial roles and expectations are strongly defined, the nursing profession may be perceived as falling short of the conventional ideals of women’s work. This can result in family members undervaluing the significance of nursing, viewing it as less respectable or desirable compared to more traditional roles.

N3: Because nursing work involves shifts, it is difficult to take care of the family, especially after having children. Many children have weekends off, but I have to go to work in the hospital.

N8: Because nurses need shifts, and the off-duty time of the operating room is uncertain, sometimes they have made an appointment with their families but can’t attend family dinners on time. Especially during long holidays, we are involved in standby classes, and it is difficult to plan long-distance trips with our partners.

(2) The enthusiasm for work has gradually waned.

Participants reported entering the nursing profession with high enthusiasm and passion, driven by a strong commitment to patient care and the desire to make a meaningful impact. However, over time, this initial enthusiasm often diminishes. The ongoing challenges, including high stress levels, demanding working conditions, and a lack of recognition for their efforts, can contribute to a gradual loss of passion for their work. When nurses feel that their work is not valued or appreciated, their initial sense of purpose and fulfilment can shift towards disillusionment and negativity. This erosion of passion can be exacerbated by factors such as insufficient support, low social status, and high turnover rates, which, in turn, affect job satisfaction and overall morale. As a result, what was once a profoundly fulfilling career can become a source of stress and frustration, highlighting the need for systemic changes to support and recognise the vital contributions of nursing professionals.

N9: When I graduated, I also worked with enthusiasm, especially at the beginning. I experienced the great rescue of patients and felt very fulfilled. However, some colleagues were beaten by the family members of the patients during the rescue process. Some family members insulted us at will. And sometimes I felt sorry for my efforts, and my work became less and less enthusiastic.

N6: The words of work enthusiasm are all deceiving young nurses with low seniority. When you have been working in a clinic for a long time, you will see a lot of life and death, and you will find that you have gradually become numb to life. I can no longer find my initial heart in this job.

### Contributing to “intent to leave”

Despite nurses’ deep appreciation for the significance of their work and its critical role in healthcare, many express a firm intention to turn over due to the increasing intensity and pressure associated with the profession. Participants consistently reported that, given the opportunity, they would consider leaving their positions or opting for a different career if they could choose again. The high levels of stress, demanding work conditions, long hours, and emotional strain contribute to this intense desire to leave the field. This dissatisfaction highlights a critical issue within the nursing profession. While nurses understand and value their essential contributions, their challenges may lead to burnout and a high turnover rate. Addressing these issues is crucial to improving job satisfaction, retaining skilled professionals, and ensuring the healthcare system functions effectively.

N14: Hospitals are semi-self-financing institutions. To ensure everyone’s income, managers will not recruit more people, so there may not be as many applicants, but the number of patients is increasing, resulting in an increased workload. I often need to work overtime. If I have the opportunity, I will consider working in other institutions.

N10: I will not support my children to study medicine, or even oppose them to engage in medical work. If I can choose again, I will never choose to be a nurse.

## Disscussion

According to participants’ statements, nursing work mainly involves two levels: “physical stigma” and “social stigma”, while nurses’ self-cognition of the occupation is not singular but contradictory, involving the coexistence of occupational identity and occupational denial. On the one hand, nurses recognise the necessity and importance of nursing work; on the other hand, they deny their work to some extent due to public misunderstanding of it.

As far as “physical stigma” is concerned, nursing work often involves handling various pollutants, such as body fluids and blood [24], which requires nurses to pay special attention to personal hygiene and protective measures at work to ensure their own health and safety. There are many risks in nursing work itself. Besides the risk of contracting infectious diseases [25] and encountering emergencies, such as providing first aid to patients [26], nurses will also face significant challenges in the nurse-patient relationship. Due to the special nature of work, nurses may face anxiety, pain and even resentment from patients and their families [27]. This requires nurses to have strong professional knowledge and emergency response skills, as well as emotional management and communication skills, to maintain a professional attitude and provide the best nursing care for patients. The hospital working environment is also full of negative energy, which may come from the pressure of patients facing illness, pain and even death [28]. Nurses need to maintain a stable state of mind in such an environment, provide care and comfort to patients, and seek ways to protect themselves and support their mental health. Compared with some occupations, nurses’ work is often passive, and they need to take appropriate actions according to the arrangement of medical teams and patients’ needs [29]. This requires nurses to respond flexibly while working closely with the medical team to ensure patients receive comprehensive care [30]. Some studies [31] have found that leadership also plays a vital role in shaping the organisational environment. They should actively advocate for a caring attitude towards employees and provide the necessary support and resources to ensure that every nurse feels respected and valued at work [32]. By establishing a positive working atmosphere, nurses’ job satisfaction and emotional stability can be effectively improved [33], thereby providing patients with higher-quality nursing services.

The word “social stigma” is no stranger to nurses. The research [34] shows that stereotypes about the nursing profession persist in society. Nursing work mainly involves assisting doctors, and its tasks are simple and often unprofessional. Therefore, many people often make negative comments about nurses, thinking that nurses are only doctors’ assistants, while ignoring their key role in patient care and medical treatment [35]. The existence of this stereotype negatively impacts nurses’ career development and mental health [36]. Although nurses play a vital role in the healthcare system, unfortunately, they are not respected and recognised as they deserve. The social status of nurses is relatively low, reflecting the cognitive biases across occupations in the medical industry [37]. For the past century, the nursing staff in China has mainly come from vocational and technical schools. With the gradual popularisation of undergraduate education in China, although the educational background structure of nursing staff has changed [38], most nursing staff have graduated from college. This is also the fundamental reason for the formation of public stereotypes about nurses, such as “low educational background” and “weak skills”. Due to the lack of recognition of nurses’ professional value, some nurses may be limited in terms of promotion or salary, which will also affect their work enthusiasm and professional satisfaction [39].

In the course of medical treatment, if results are unsatisfactory, nurses can easily become the object of criticism and may even bear responsibility for it [40]. With the popularity of social and online media, information spreads rapidly, easily leading to an upsurge in public sentiment [41], making the nurse-patient relationship more tense. Nurses may be scolded and made difficult by patients or their families. In this case, nurses can only choose to submit to humiliation because they want to resolve conflicts with a professional and mature attitude, and at the same time, they don’t want the situation to expand or affect the normal medical treatment of other patients. This situation may lead nurses to feel depressed and stressed, and even doubt their career choices [42]. However, it is important to realise that nurses’ work is more complex than it seems. They bear the important responsibility for patients’ lives and health and provide comprehensive care through superb medical skills and warm care [43]. To address this cognitive bias regarding social status, we need to enhance the public’s understanding of and respect for nurses, thereby fostering a more just, respectful, and supportive social environment for nurses.

dentity can be seen as a structure that combines one’s own values and beliefs with social and professional ones. In most definitions, professional identity is grounded in values and ethics [44], especially in nursing. Professional identity is a crucial link for nurses [45]. It is not only the pride and identity of their profession, but also the sense of responsibility and mission in their work. Nurses bear the responsibility for patients’ health and life at every stage of their work, which gives them a strong sense of identity with the profession [46]. Highly technical features make nurses indispensable members of the medical team and help them realise their important role in the medical environment. With advances in medical technology and services, the importance of nursing work cannot be ignored. Nurses play a key role in disease prevention, health education, and patient care [47]. They are the link to patients in the medical system and an important safeguard for the quality of medical services. In addition, the stability of nursing work is also attractive. The medical industry has always been a relatively stable field [48]. With the ageing population and rising medical demand, the nursing profession will continue to grow, providing a relatively safe and stable professional environment for practitioners [49]. It enables nurses to plan and develop their careers over the long term.

However, recognition of the nursing profession may be denied in certain cases. This may stem from a misunderstanding or an insufficient understanding of nurses’ duties [50]. Some people may think that nurses’ job is only to follow doctors’ instructions, while ignoring nurses’ unique contributions in patient care, health education and so on. At the same time, family recognition is also an important aspect. If family members do not understand nurses’ duties and responsibilities, it may lead to the devaluing of nurses’ work or a lack of support [51]. In this case, nurses may find it difficult to communicate and explain, and they need to make greater efforts to earn their families’ understanding and support. This may lead to a strong willingness to leave [52], as they may start to consider seeking a more recognised and ideal working environment to realise their career ambitions. Nurses may have their own career pursuits and ideals [53], but the actual work situation does not align with them. This leaves nurses feeling disappointed and confused, and they may need to consider how to adjust their career plans and goals.

In this case, we need to break these stereotypes and make society more aware of and more respectful of the importance of the nursing profession. Schools, hospitals, and the media can convey the professionalism and importance of nurses to the public through publicity, education, professional lectures, and other means. In addition, it is necessary to strengthen vocational training and development for nurses, so that they can grow in their professional fields and demonstrate professional standards and abilities. At the same time, you can communicate with your family, share your love and dedication to the nursing profession, and strive for their understanding and support. In this way, we can truly realise the due respect and recognition for nurses, thus improving the service quality of the whole medical and health system.

### Limitation

While the primary focus of this study is to understand the work experiences of clinical nurses and their perceptions of occupational stigma, it’s important to acknowledge its limitations. The study investigated the work experiences of nursing staff, but the diverse range of departments from which participants were drawn led to wide variation in experiences, potentially affecting the study results. It’s important to note that universality is not the primary aim of this study or the central proposition of stereotype research. This study should note that the sample included one director of nursing (N14), who is also a doctoral student. As a senior manager, her professional perspective differs significantly from that of frontline clinical nurses; she may be more inclined to describe “dirty work” from the perspective of managerial responsibilities and institutional frameworks rather than from the perspective of physical labour and emotional experiences, which may, to some extent, influence how the data reflect actual clinical experiences. This perspective should be viewed as a supplement at the organisational level, rather than as the general stance of frontline nurses. Future research may consider stratified sampling to further examine the impact of professional hierarchy on narratives.

## Conclusion

This study investigated the work experiences of nurses with a Chinese background and found that they were generally strongly influenced by professional stigma. At the same time, we find that nurses’ professional identity is not single but contradictory, a broader focus than previous studies. In addition, we found that the workers’ misunderstanding of nursing work is the main reason for nurses’ professional stigma, and nursing managers and the government can convey the professionalism and importance of nurses to the public through various channels.

## Data Availability

Due to the sensitive nature of the questions asked in this study, survey respondents were assured raw data would remain confidential and would not be shared.
